# Real‐time high spatial resolution dose verification in stereotactic motion adaptive arc radiotherapy

**DOI:** 10.1002/acm2.12364

**Published:** 2018-06-05

**Authors:** Mitchell Duncan, Matthew K. Newall, Vincent Caillet, Jeremy T. Booth, Paul J. Keall, Michael Lerch, Vladimir Perevertaylo, Anatoly B. Rosenfeld, Marco Petasecca

**Affiliations:** ^1^ Centre for Medical Radiation Physics University of Wollongong Wollongong NSW Australia; ^2^ Northern Sydney Cancer Centre Royal North Shore Hospital St. Leonards NSW Australia; ^3^ Institute of Medical Physics School of Physics University of Sydney NSW Australia; ^4^ Radiation Physics Laboratory School of Medicine University of Sydney NSW Australia; ^5^ SPA‐BIT Kiev Ukraine

**Keywords:** 2D silicon array, MLC tracking, QA of adaptive radiotherapy, small field dosimetry

## Abstract

**Purpose:**

Radiation treatments delivered with real‐time multileaf collimator (MLC) tracking currently lack fast pretreatment or real‐time quality assurance. The purpose of this study is to test a 2D silicon detector, MagicPlate‐512 (MP512), in a complex clinical environment involving real‐time reconfiguration of the MLC leaves during target tracking.

**Methods:**

MP512 was placed in the center of a solid water phantom and mounted on a motion platform used to simulate three different patient motions. Electromagnetic target tracking was implemented using the Calypso system (Varian Medical Systems, Palo Alto, CA, USA) and an MLC tracking software. A two‐arc VMAT plan was delivered and 2D dose distributions were reconstructed by MP512, EBT3 film, and the Eclipse treatment planning system (TPS). Dose maps were compared using gamma analysis with 2%/2 mm and 3%/3 mm acceptance criteria. Dose profiles were generated in sup‐inf and lateral directions to show the agreement of MP512 to EBT3 and to highlight the efficacy of the MLC tracking system in mitigating the effect of the simulated patient motion.

**Results:**

Using a 3%/3 mm acceptance criterion for 2D gamma analysis, MP512 to EBT3 film agreement was 99% and MP512 to TPS agreement was 100%. For a 2%/2 mm criterion, the agreement was 95% and 98%, respectively. Full width at half maximum and 80%/20% penumbral width of the MP512 and EBT3 dose profiles agreed within 1 mm and 0.5 mm, respectively. Patient motion increased the measured dose profile penumbral width by nearly 2 mm (with respect to the no‐motion case); however, the MLC tracking strategy was able to mitigate 80% of this effect.

**Conclusions:**

MP512 is capable of high spatial resolution 2D dose reconstruction during adaptive MLC tracking, including arc deliveries. It shows potential as an effective tool for 2D small field dosimetry and pretreatment quality assurance for MLC tracking modalities. These results provide confidence that detector‐based pretreatment dosimetry is clinically feasible despite fast real‐time MLC reconfigurations.

## 1 INTRODUCTION

Stereotactic body radiation therapy (SBRT), stereotactic ablative radiotherapy (SABR), and stereotactic radiosurgery (SRS) are highly conformal radiotherapy modalities for small tumors and functional abnormalities. They use a high dose per fraction (from 3 to 20 Gy) and fewer fractions than conventional external beam radiotherapy (EBRT) over the course of treatment. Field sizes are also typically smaller than conventional radiotherapy, commonly less than 4 × 4 cm^2^, where effects such as the loss of charged particle equilibrium (CPE) and partial source occlusion are present.

SABR is often used to treat early‐stage lung tumors.^1^ Patient respiration has been reported to affect target position by up to 30 mm during EBRT.^2^ Intrafraction tumor motion can have a considerable impact on the dose delivered to the tumor volume and surrounding healthy tissue,^3^ if motion is not compensated. Careful quality assurance processes are essential in the application of such highly conformal radiotherapy modalities when tumor motion is present.^4^

Intrafraction tumor motion can be accommodated in the treatment planning process using an extra margin called the internal target volume (ITV). This margin encompasses the entire volume occupied by the moving tumor throughout several breathing cycles, obtained from 4DCT data. Inclusion of an ITV generally leads to a larger planning target volume (PTV) and as a result, there is often more healthy tissue irradiated compared to a treatment experiencing minimal tumor motion. Another approach to manage organ motion is gating the beam to treat only when the tumor is in a particular position.^5^, ^6^, ^7^

Real‐time adaptive radiotherapy (ART) aims to treat the target volume with sensible margin reduction, in comparison to an ITV contouring, by monitoring the position and shape of the tumor continuously throughout the treatment in order to modify the beam in real time.^8^

The aim is to reduce the size of the ITV considerably, so that healthy tissue receives minimal dose, while at the same time delivering the correct dose to the target to achieve the highest possible therapeutic outcome.

Kilovoltage Intrafraction Monitoring (KIM) is one real‐time monitoring technique^9^; it uses the gantry equipped kV x‐ray imager to monitor the position of the tumor in real time during treatment. An algorithm calculates the difference in tumor position from the current and previous image to predict the target position in 3D. When the tumor moves outside a predefined tolerance, the treatment is paused and the patient is repositioned through use of couch motion prior to resuming treatment.

There are several other possible strategies to manage patient motion during EBRT, including pretreatment and intratreatment imaging, respiratory control devices or robotic couches.

Recently, an extensive review was published of current motion management strategies applied in the clinic.^10^

An alternative motion management approach in real‐time ART uses Calypso, the electromagnetic guided tumor tracking adopted by Varian, and real‐time beam modulation using the multileaf collimator (MLC). The Calypso system detects and reconstructs the position of electromagnetic beacons implanted in proximity of the tumor volume.^11^, ^12^

The combination of Calypso and MLC tracking has been implemented clinically at Royal North Shore Hospital (Sydney–Australia) as a real‐time tumor monitoring solution for ART. In 2013, MLC tracking was used for the first time during a prostate VMAT treatment^13^ and more recently with lung SABR.^14^ In that study, the dose distribution was recalculated on the patient CT dataset using a motion encoded treatment plan derived from analysis of the treatment log files (dynalog). Results showed a reduction in the PTV size by up to 40% and a reduction of mean dose to normal lung tissue of 30%, compared to a standard ITV‐based delivery.^14^

Tracking techniques such as KIM and radiofrequency tracking use feedback systems that may induce a signal in the chosen dosimeter, i.e., kV x rays and RF electromagnetic radiation, respectively. It is important that the dosimeter used during real‐time ART is also unperturbed by these additional signals and only measures the dose due to the MV treatment.

Small radiation fields introduce some extra dosimetry requirements compared to larger fields. Namely high spatial resolution of the detector array for use in steep dose gradients as well as small sensitive volume of a single detector, compared to beam size, to avoid volume averaging. The adopted dosimeter system should ideally be close to tissue equivalent, so as not to perturb the beam.^15^, ^16^

There are several dosimeters that can potentially be used for small field dosimetry, each with their advantages and disadvantages.

EBT3 film and Fricke or polymer‐based gel have the required spatial resolution for small field dosimetry. They can give 2D and 3D dose information, respectively; however, they lack a real‐time readout.^16^, ^17^

Ionization chambers can be read out in real time and can be calibrated for absolute dose; however, their spatial resolution and volume averaging effects can limit their use in high‐resolution arrays for small field dosimetry.^16^

Silicon diodes can be fabricated with a small sensitive volume; this means they can form 2D arrays with high spatial resolution. The density of silicon is considerably higher than that of water, so energy dependence needs to be characterized as well as dose rate and angular dependence.^18^

Log file analysis is a reasonable machine QA tool for MLC and gantry positional errors. However, it is unable to provide information on the position of a moving phantom relative to the beam or independently evaluate the performance of an external tracking system, such as Calypso.

The Centre for Medical Radiation Physics (CMRP) at University of Wollongong has developed a 2D monolithic silicon diode array detector to be used for QA of small field real‐time ART: MagicPlate‐512 (MP512). It has been designed to independently monitor external beam radiotherapy treatments under complex clinical conditions and reconstruct dose distributions in real time. It is capable of pulse‐by‐pulse monitoring of the linac beam and has a spatial resolution suitable for small field dosimetry. It has been rigorously characterized for basic square fields in terms of percentage depth dose, output factor, beam profiles, dose per pulse, and dose linearity.^19^ It has also been used with modulated IMRT fields, with a fixed gantry angle and a single patient motion applied.^20^ This study shows dosimetric results from MP512 using a two‐arc VMAT delivery with realistic patient motions and tracking by the means of the Calypso localization system. Dose was delivered to a solid water phantom for three different patient motion traces obtained by 4DCT at Royal North Shore Hospital (Sydney–Australia). Dose maps obtained from MP512 were compared to EBT3 film and treatment planning system calculations.

## 2 MATERIALS AND METHODS

MP512 was placed in a solid water phantom on top of a movable platform. Three patient motion traces were supplied to the platform to simulate clinical lung motion during treatment delivery. Calypso (Varian Medical Systems, Palo Alto, CA, USA) was used to monitor the position of the phantom and real‐time MLC tracking was used. A two‐arc VMAT plan was delivered for various motion modalities. Dose was monitored in real‐time with MP512 and compared to EBT3 film and the treatment planning system. Each component of the experiment is described in more detail below.

### 2.A MP512 detector array

**Figure 1:**
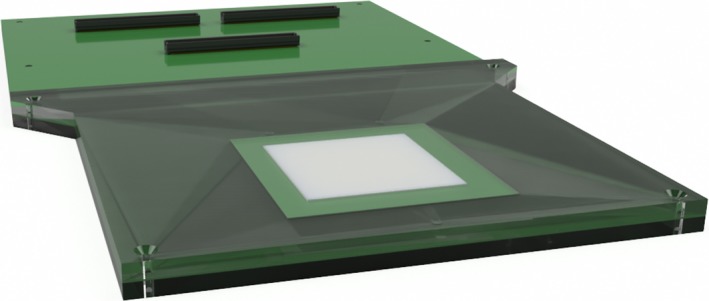
Rendering of MP512 detector mounted on printed circuit board (PCB) and encapsulated in a PMMA phantom.

MP512 (Figure 1) features a 52 × 52 mm^2^ array of 512 individual ion implanted silicon diodes on an epitaxial p‐type silicon substrate. Each diode has a sensitive area of 0.5 × 0.5 mm^2^ with 2 mm center‐to‐center distance. The detector is assembled on a 500 *μ*m thick printed circuit board which allows for mechanical rigidity and connection of the readout electronics.

MP512 is a monolithic silicon detector which has an intrinsic asymmetric structure creating an angular dependence, which needs to be considered. Angular correction factors for 6 MV photons on a Varian 2100iX linac have previously been obtained.^21^ The correction is valid for all MP512 devices; individual diode sensitivity is corrected pixel‐by‐pixel by a flat field prior to measurements. The rotation of the linac was independently monitored by an inclinometer positioned on the gantry and synchronized with the data acquisition system. This allowed for on‐line correction of the detector response frame‐by‐frame.

To calibrate the detector, a dose linearity measurement in the dose range 50 cGy to 1000 cGy was delivered at d_max_ in solid water for a 10 × 10 cm^2^ field size with 6 MV photons from a Varian 2100iX linac. Detector charge (nC) was plotted against dose (cGy) and the slope of the linear relationship was used to define the dose calibration factor.

The data acquisition system (DAQ) of the detector comprises of an analog front end (AFE) connected to an analog to digital converter. These are controlled by a field programmable gate array (FPGA) which also provides the de‐randomization of the detector channels, acquisition of the data from the inclinometer and data packing for the USB2.0 interface used to communicate with a standard laptop. The AFE is a commercial chip from Texas Instruments (Dallas, USA), the AFE0064, and features 64 current integrators whose output is proportional to the charge collected on a variable capacitor over a user‐defined integration time window. The DAQ features eight AFE chips, which are parallelized to readout the entire detector at a maximum frame frequency of 7 kHz. The system can be synchronized with the linac electron gun pulse, so that current is integrated only when the beam is on, minimizing noise and allowing for measurement of the dose delivered by every linac pulse. Full specifications and benchmarking of the DAQ have been published by Fuduli et al.^22^

A software control panel for the instrument was designed in house using the Qt Creator Integrated Development Environment (IDE) (The Qt Company — Espoo, Finland) based on C++. It manages all communication with the FPGA including definition of the integration time, dynamic range, acquisition time, and frequency of measurements. It also provides real‐time visualization of the detector response and allows for retrospective analysis of the data acquired.

### 2.B Reproducing organ motion

The patient lung motions were simulated using the 6D Hexamotion movable platform by ScandiDos (Uppsala, Sweden) which provides simultaneous motion in each of the x (left‐right), y (sup‐inf), and z (ant‐post) directions. Motion patterns were supplied by Northern Sydney Cancer Centre (Royal North Shore Hospital, St Leonards NSW) and were extracted from patient 4DCT data. For this study, three motion patterns were used:

1. Patient 1: Lung trace with approximately 8 mm sup‐inf offset
2. Patient 2: Lung trace centered around zero (null sup‐inf offset)
3. Patient 3: Lung trace with a predominant component in the lateral direction.

These motion traces were chosen as they represent three unique types of respiration.

**Figure 2:**
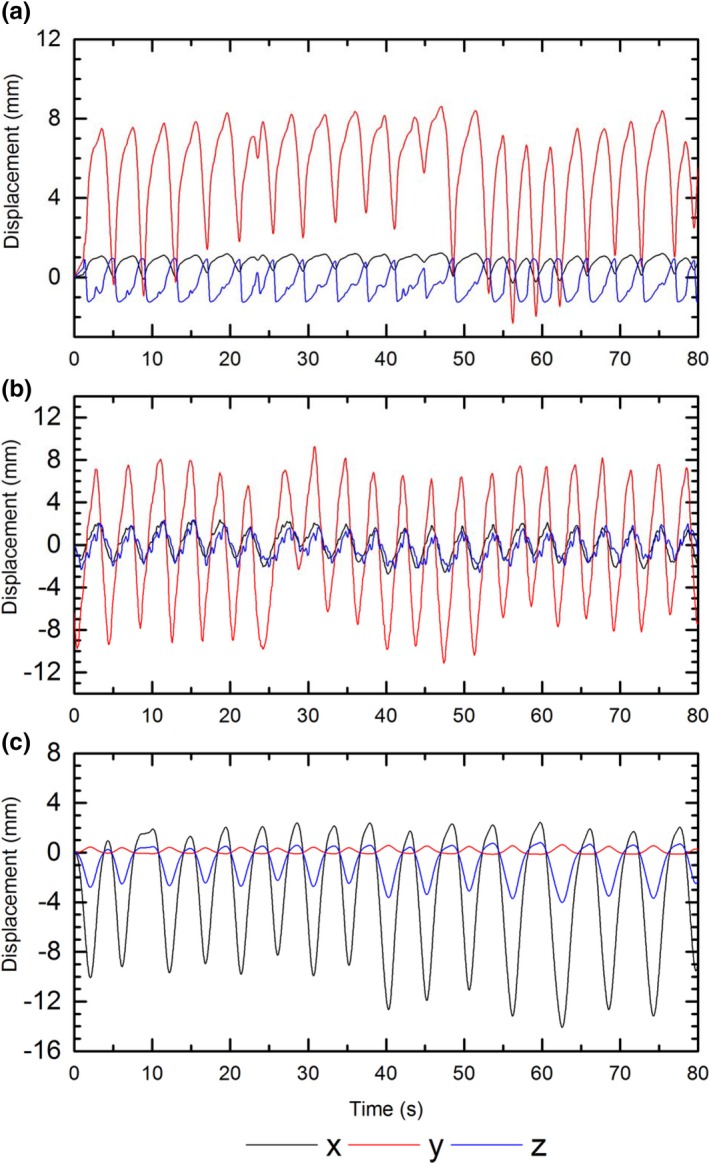
Three‐dimensional lung motion traces corresponding to (a) Patient 1, (b) Patient 2 and (c) Patient 3. Data are from 4DCT sampled every 25 ms. X component corresponds to motion in left‐right direction, Y component corresponds to motion in sup‐inf direction, and Z corresponds to motion in ant‐pos direction.

Figure 2 shows the motion traces as a function of time for each patient in x, y, and z directions for approximately 80 s, corresponding to the time required to deliver the planned dose to the target.

### 2.C Motion tracking

MLC tracking is performed using the Calypso 4D localization system (Varian Medical Systems — Palo Alto, CA, USA) for real‐time monitoring of the target motion in conjunction with an MLC driving software developed at University of Sydney.^23^ Electromagnetic beacons are used to determine the target position, this information is used by the tracking software to signal the MLC controller to configure the field shape in response to the detected motion of the target.^13^ Two different algorithms were input to the MLC controller: passive feedback and predictive feedback. The passive feedback algorithm calculates the position of the moving target based on information from Calypso, and instructs the MLC controller to reconfigure the beam aperture accordingly. There is a measurable latency of approximately 230 ms 13 between detection by Calypso and actuation of the MLC leaves due to computation time and finite leaf speed, meaning that the beam lags behind the real target trajectory. The predictive feedback algorithm uses kernel density estimation^23^ and a short learning time frame in order to approximate the position of the target, based on the difference between nominal and effective tumor position. By predicting the target position at a time of T+τ (where T is current time and τ is a small increment of time) and setting τ=230ms, the system latency is almost eliminated.^20^, ^24^

Four tracking modalities were used to study the effect of motion on the dose distribution:

1. No motion: phantom is static throughout the treatment delivery
2. Motion with no tracking: the phantom follows the supplied motion trace and no MLC tracking is applied
3. Motion with passive tracking: the supplied motion trace is adopted by the phantom and the MLC motion is controlled by Calypso
4. Motion with predictive tracking: the phantom follows the supplied motion trace, Calypso tracking is activated and the predictive algorithm is used^23^ to minimize the delay between detected motion and the corresponding MLC movement.

### 2.D Detector encapsulation and phantom

MP512 was encased between two 5 mm thick sheets of PMMA for structural rigidity (see Figure 1). There is an air gap above the sensitive volume to match the detector response to that of EBT3 film for small field beams down to 1 × 1 cm 19, 25 and to minimize the density and perturbation effects of silicon.^26^, ^27^

An aluminum shielding box of 2 mm thickness was designed to mitigate the baseline fluctuations due to the electromagnetic (EM) field generated by Calypso.^20^ The time‐varying EM field induced a current in the wiring and DAQ electronics, which contributed to fluctuations of the baseline signal (noise). The aluminum box covered and sealed the entire detector and data acquisition system. Its effect on dosimetry has been investigated by the means of Geant4 and proved experimentally to have negligible effects at 1.5 cm water equivalent depth and below.^20^ The phantom geometry is such that the minimum thickness of solid water in the beams eye view is 1.5 cm throughout each arc.

The aluminum box was designed to allow 5 mm of solid water to be placed on top of the detector phantom, producing a water equivalent depth of approximately 1.5 cm, to simulate for example a chest wall. The calculation of the water equivalent depth of the detector (with beam normally incident to phantom surface, gantry at zero) is shown in Eq. [Link] where $t_{i}$ and $\rho_{i}$ are the thickness and density of each of the materials, respectively. Data used in the calculation are listed in Table 1.

**Table 1:** Data for calculating water equivalent depth of the detector sensitive volume

Material	Thickness ($t_{i}$) mm	Density ($\rho_{i}$) g/cm^3^
PMMA	4	1.18
Solid Water	5	1.03
Aluminum	2	2.7

$$\begin{array}{cc} & d=\sum_{i}t_{i}\times \rho_{i}=\;15.27\;mm\\ & Equation\;1\;\;:Calculation\;of\;water\;equivalent\;depth \end{array}$$

**Figure 3:**
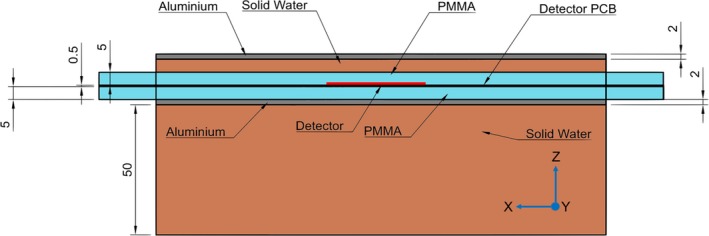
Cross‐section of scattering conditions of the solid water phantom. Units are mm. Detector surface is at an approximate water equivalent depth of 1.5 cm and the solid PMMA phantom is encapsulated by aluminum to minimize induced noise from RF field of Calypso. Coordinate system is marked with the Y direction coming out of the page.

The aluminum box was placed above 5 cm of solid water, figure 3 shows the cross‐section of the detector phantom with the aluminum sheets and solid water blocks in place.

### 2.E Treatment planning and delivery

**Figure 4:**
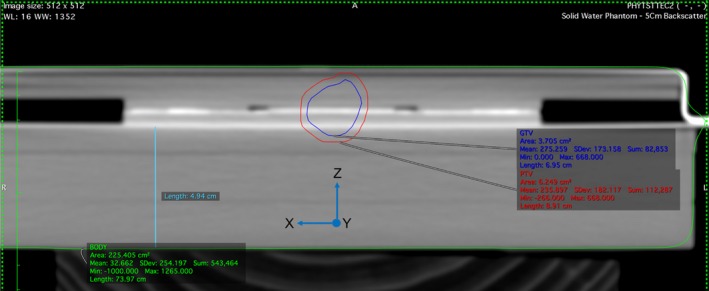
GTV (blue) and PTV (red) margins defined on a CT dataset of the detector phantom represented in a transverse plane view. Directions are indicated with Y (sup‐inf) coming out of page.

Two VMAT arcs were used to deliver 496 MU and 508 MU at 6 MV on a Varian Clinac iX. The first arc started at gantry angle 150 degrees, rotated in a counter‐clockwise direction and stopped at 340 degrees, the second arc started at 340 degrees, rotated clockwise and returned to 150 degrees. This beam arrangement allowed sufficient target coverage while avoiding the treatment couch. A gross tumor volume (GTV) was contoured manually around a hypothetical spherical target of 2 cm diameter. The target's center was aligned with the central pixel of the detector. A PTV was defined as a 3 mm expansion of the GTV to create a clinically relevant lung plan while allowing the contours to remain within the phantom geometry. The Eclipse Treatment Planning System (TPS) (Varian Medical Systems — Palo Alto, CA, USA), at Northern Sydney Cancer Centre was used to generate a treatment plan which was inversely calculated on the CT dataset of the phantom (Figure 4). A dose prescription of 5 Gy in a single fraction was implemented. CT slice thickness was 1 mm and dose was calculated using a 2‐mm dose grid. No density overrides were applied as there were no appreciable artifacts in the dataset. Note that the same plan was delivered for all motion modalities, i.e., there was no ITV‐based plan used for comparison. The aim of this work was to characterize MP512 for use in adaptive arc modalities using MLC tracking and gantry rotation. There was no intention to comment on the effectiveness of MLC tracking versus ITV‐based planning in terms of clinical outcomes; hence, the same plan was used for all modalities.

**Figure 5:**
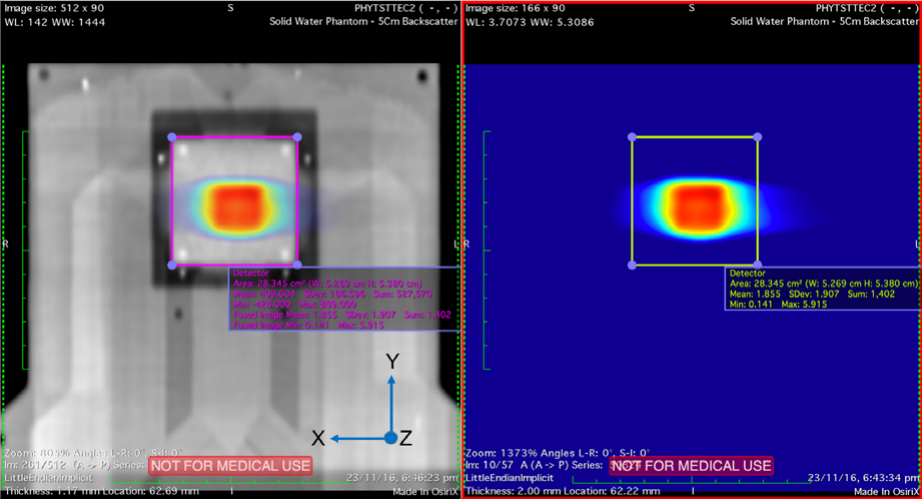
2D dose map extracted from Eclipse TPS overlaid on CT slice of MP512. Boxes highlight the sensitive area of the detector and represent the dose map region used for gamma analysis comparison (coronal plane views).

In Figure 4, a slice of the CT dataset incorporating the GTV and PTV margins is shown. Figure 5 depicts the 2D dose map from Eclipse overlaid on MP512 from a beam's eye view (coronal plane). The square regions overlaid on Figure 5 highlight the sensitive area of the detector and correspond to the dose map position extracted for comparison with the experimental results.

### 2.F Dosimetric measurements

EBT3 film was used to verify dose accuracy utilizing a gamma analysis of the 2D dose distributions. To coregister the dose maps, film sheets were sliced to the dimensions of the silicon detector and inserted into the PMMA phantom. The airgap recess in the PMMA phantom was common to both the detector and film, so dose maps were intrinsically coregistered. MATLAB was used to convert the optical density of the films to dose using a set of calibration films.^21^ Local gamma analysis was performed using a MATLAB script. Gamma pass rates are reported for 2%/2 mm and 3%/3 mm dose difference and distance to agreement criteria. The same method was adopted to compare MP512 dose maps to the extracted 2D dose maps from the TPS. Finally, a comparison was made of MP512 dose maps for all motion modalities with respect to the no‐motion case to quantify the accuracy in reconstructing the effects of MLC tracking.

Dose profiles in the sup‐inf and left‐right (lateral) directions for MP512 and EBT3 were plotted on the same axis for each motion modality. The profiles were fitted with a shape preserving polynomial interpolation in MATLAB and aligned to 50% of the central axis dose response. The fit allows for accurate calculation of the full width at half maximum (FWHM) and left and right penumbral width, i.e., width of the profile between 80% response and 20% response. The fitting step used is 20 *μ*m and the estimated accuracy for determination of the FWHM and penumbra width is approximately ±0.25 mm. This corresponds to physical size of a single silicon diode, which is approximately 0.5 × 0.5 mm^2^.

The baseline noise of the detector signal was determined to be approximately ±1%, the dose uncertainty of the EBT3 film was calculated^19^ to be approximately ±2% and error bars are reported on the dose profiles, accordingly.

The effect of motion on dose response was analyzed by plotting the point‐to‐point dose difference to show the areas of under and over dose and to highlight the effect that each tracking modality had on reducing these errors.

## 3 RESULTS

### 3.A Dose linearity

**Figure 6:**
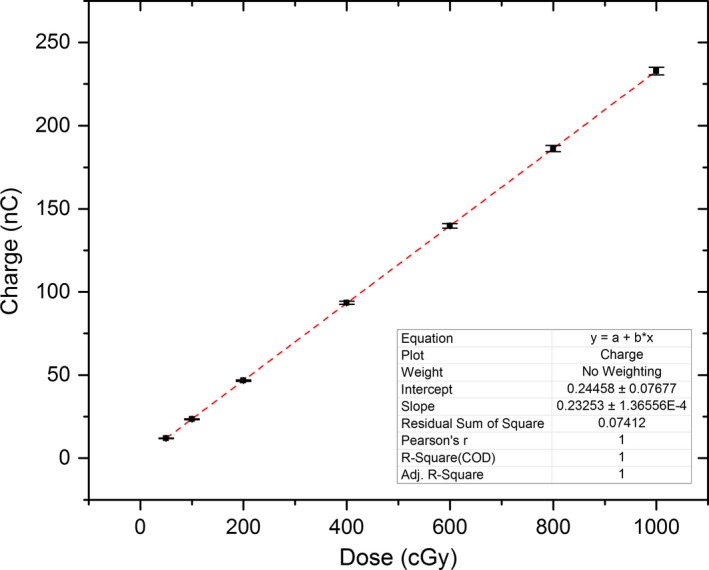
Linear dose response exhibited by MP512 for cumulative dose delivery. The slope of the linear fit is used to convert measured detector charge (nC) to absorbed dose (cGy). Error bars indicate measured baseline fluctuation of 1%.

The response of MP512 to accumulated dose is shown in Figure 6. The relationship of charge to dose in the 50 to 1000 cGy range is linear. The MP512 measured charge is converted to dose using slope of the linearity curve.

### 3.B Gamma analysis

**Figure 7:**
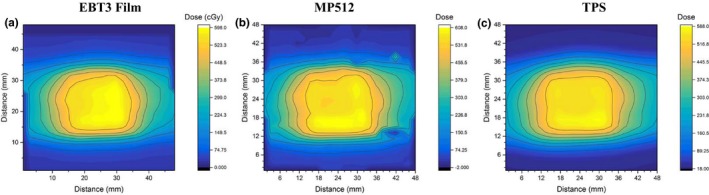
2D integral dose maps reconstructed from (a) EBT3, (b) MP512 and (c) TPS for the no‐motion case.

The integral dose maps obtained from EBT3, MP512, and TPS are shown in Figure 7. Angular corrections were applied frame‐by‐frame to the MP512 response before integrating the dose.

The gamma pass rates comparing EBT3 to MP512 and TPS to MP512 for the no‐motion case are shown in Table 2. Table 3 shows the gamma analysis results for the three motion patterns in respect to the no‐motion case as measured by MP512.

**Table 2:** Gamma pass rates for MP512 compared to TPS and EBT3. Dose maps used correspond to the no‐motion case

Tracking modality	Comparison	2%‐2mm (%)	3%‐3mm (%)
No‐motion	TPS to MP512	98.44	100
	EBT3 to MP512	95.31	99.31

**Table 3:** Gamma pass rates for MP512 vs MP512 response with respect to the no‐motion case for each motion modality

Motion trace	Tracking modality	2%‐2mm (%)	3%‐3mm (%)
Patient 1	No tracking	78.65	98.44
	Passive	98.96	99.83
	Predictive	98.44	99.65
Patient 2	No tracking	55.56	75.35
	Passive	94.62	97.05
	Predictive	96.01	98.09
Patient 3	No tracking	79.69	91.32
	Passive	95.66	98.61
	Predictive	93.23	96.35

### 3.C Dose profiles measured by MP512 and EBT3 film

**Figure 8:**
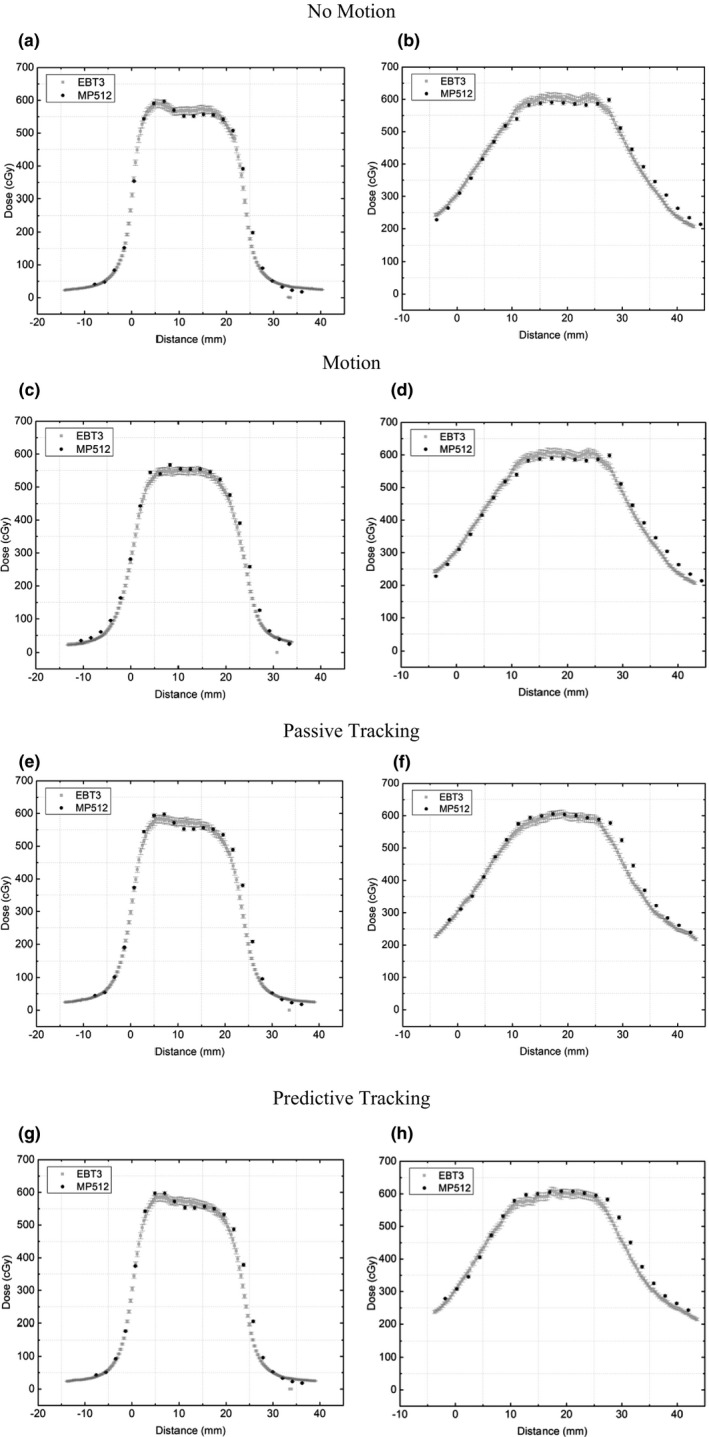
MP512 vs EBT3 Dose Profiles for various motion modalities with Patient 1 motion applied. Left Column: Profiles in the sup‐inf direction. Right Column: Profiles in the left‐right direction.

In Figure 8, the dose profiles are illustrated in the vertical and horizontal directions of MP512 and EBT3 film for each of the motion modalities. The uncertainty in EBT3 film measurement was calculated^19^ to be ±2% and is within the size of the data points. Analysis of the baseline fluctuation of MP512 produces an uncertainty of ±1%. Table 4 summarizes the analysis of the FWHM and penumbral width for the dose profiles for the sup‐inf direction.

**Table 4:** Summary of the comparison of FWHM and left and right penumbral width between MP512 and EBT3 for dose profiles in the vertical direction

Modality	Detector	FWHM (mm) ±0.3 mm	Left Penumbra (mm) ± 0.3 mm	Right Penumbra (mm) ± 0.3 mm
No‐motion	EBT3	23.9	3.7	4.2
	MP512	24.5	4.1	4.7
	TPS	23.7	4.1	4.7
Motion	EBT3	24.0	5.3	5.4
	MP512	24.6	5.7	5.9
	*Difference*	0.6	0.3	0.5
Passive tracking	EBT3	23.8	4.7	5.3
	MP512	24.6	4.7	5.3
	*Difference*	0.8	0	0
Predictive tracking	EBT3	23.8	4.3	5.3
	MP512	24.7	4.4	5.4
	*Difference*	0.9	0.1	0.1

### 3.D MP512 motion profiles

**Figure 9:**
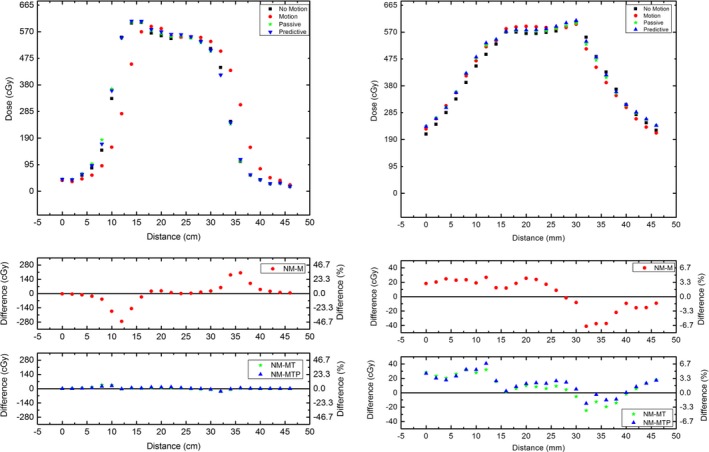
Dose profiles measured with MP512 when the Patient 1 motion was applied for each motion modality, with dose error. Left corresponds to Sup‐Inf direction and right corresponds to Left‐Right direction. In the error plots, NM corresponds to no motion, M to motion, MT to motion tracking (i.e., passive algorithm) and MTP to motion tracking with prediction.

**Figure 10:**
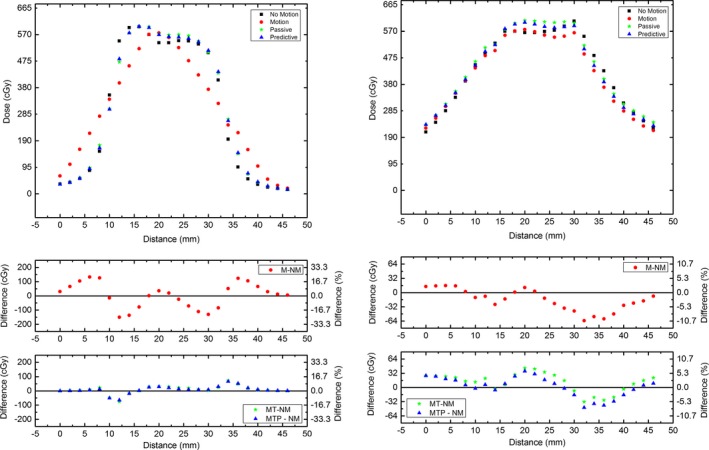
Dose profiles measured with MP512 when the Patient 2 motion was applied for each motion modality, with dose error. Left corresponds to vertical direction and right corresponds to horizontal direction. In the error plots, NM corresponds to no motion, M to motion, MT to motion tracking (i.e., passive algorithm) and MTP to motion tracking with prediction.

**Figure 11:**
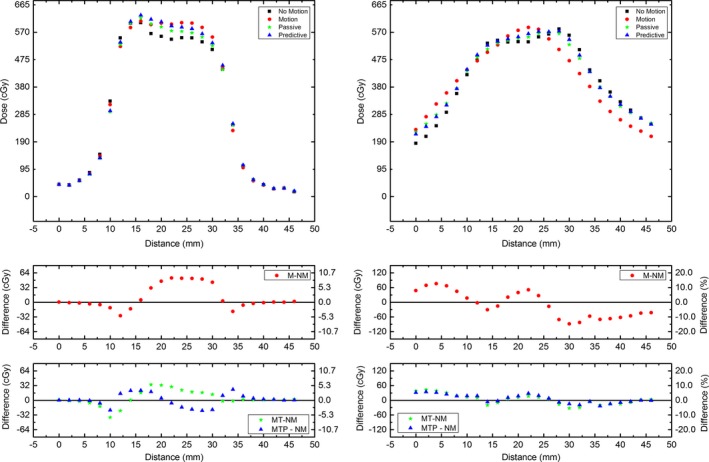
Dose profiles measured with MP512 when the Patient 3 motion was applied for each motion modality, with dose error. Left corresponds to vertical direction and right corresponds to horizontal direction. In the error plots, NM corresponds to no motion, M to motion, MT to motion tracking (i.e., passive algorithm) and MTP to motion tracking with prediction.

The profiles shown in Figure 9 highlight the effect of Patient 1 motion on the dose distribution. The profiles are reconstructed from MP512 for each motion modality. They are plotted on a single axis and the point‐to‐point dose difference is included in both cGy and percentage to highlight areas of under and over dosage. The dose difference is referenced to the baseline no‐motion case. The same profiles are generated for Patients 2 and 3 in Figs 10 and 11, respectively.

## 4 DISCUSSION

Gamma analysis of the 2D dose maps from MP512 showed a very close match to the 2D dose measured from EBT3 film and dose extracted from Eclipse TPS. Using a 2%/2 mm acceptance criteria, the response of MP512 matched that of EBT3 for 95% of points and TPS for 98% of points. When a 3%/3 mm acceptance criterion was used, the agreement was 99% and 100% for EBT3 and TPS, respectively. This confirms the accuracy of MP512 as a 2D dosimetry system even under complex clinical conditions. MP512 can reconstruct the dose delivered during realistic patient breathing with real‐time MLC tracking and full arc delivery. As a result, MP512 can be used as a tool to quantify the efficacy of a particular tracking regime, and in patient‐specific pretreatment QA.

Gamma analysis was also performed on the MP512 measured dose maps to intercompare each motion modality, this gives some insight into the effect of MLC tracking.

Patient 1 motion (with no tracking) resulted in a pass rate of 78% (2%/2 mm); however, the two tracking modalities brought the pass rate to 95% or above for both 3%/3 mm and 2%/2 mm acceptance criteria.

Patient 2 uncorrected motion had a pass rate of 55% (2%/2 mm) and Patient 3 had 80% pass rate (2%/2 mm) with motion. When tracking was applied, pass rates of above 93% were observed for both Patient 2 and Patient 3 for both criteria.

Gamma pass rates above 95% for 3%/3mm agreement are commonly accepted as sufficient to proceed with treatment.^28^ In our case when the Patient 1 motion pattern was introduced, there was a large drop in the 2%/2 mm pass rate when no tracking is applied; however, the 3%/3 mm criteria was relatively unchanged at 98%. If a gamma analysis using 3%/3mm acceptance criteria was the only form of pretreatment QA for this plan, it appears to be clinically acceptable. However, it is somewhat unlikely as this plan was designed to be used in conjunction with MLC tracking and would not be used clinically when no tracking is to be applied, rather an ITV‐based plan would be created. This result still highlights the need to use caution when depending solely on gamma analysis (especially with a rather loose acceptance criterion) as a form of pretreatment QA. A similar conclusion was reached in previous studies^29^, ^30^ which showed that gamma analysis pass rates for 3%/3 mm and 2%/2mm acceptance criteria for particular 2D dose planes had very little correlation to errors in clinically relevant patient DVH metrics and even a 1%/1mm acceptance criteria only showed moderate correlation.

MP512 monitors the beam in real time, for every linac pulse. As a result, the data are available immediately after the measurement. This information can be used to give insight into the efficacy of a particular tracking regime. For example, the results from the gamma analysis show the predictive tracking algorithm did not always lead to a higher pass rate when compared to passive tracking, in this case it was particularly dependent on the type of motion being experienced by the target volume.

In our study, for periodic motion patterns, the predictive algorithm had superior performance and gave better target tracking; however, for motion patterns that are aperiodic and erratic the passive tracking gave better results. We hypothesize this is due to the difficulty in accurately predicting an irregular signal and depends on the type and duration of the learning process adopted in the predictive algorithm.^31^ Some algorithms use an initial learning window before treatment where the program learns the breathing trace over a few respiratory cycles and others employ a dynamic type of learning which can adapt faster to irregularities in the motion.^32^

Dose profiles from MP512 and EBT3 film undergoing Patient 1 motion were plotted on a single axis and compared (Figure 8). It was found that in the sup‐inf direction (major axis of organ motion due to the breathing cycle), the penumbral width of both profiles was within 0.5 mm and FWHM agrees within 1 mm for all deliveries. The agreement of data between MP512 and EBT3 film over multiple datasets further proves the accuracy of MP512 for use in adaptive therapy treatments.

MP512 measured dose profiles from each motion modality were plotted on a single axis, this allowed a qualitative overview of the effect of patient motion and MLC tracking on the dose delivered. The effect of Patient 1 (Figure 9) motion on the dose distribution can clearly be identified through the spread of the profile and loss of the dose distribution shape presented in the no‐motion case. The discrepancy is quantified by an increase in left penumbral width of 1.62 mm compared to the no‐motion case. Passive tracking was able to reduce the penumbral width by 60%. The predictive algorithm showed a reduction of 79%, an agreement within 0.34 mm of the no‐motion case. The motion trace for Patient 1 is primarily in the vertical direction with a maximum lateral displacement of approximately 1.5 mm (Figure 2a); because the lateral shift is smaller than the MLC leaf width, it produces very little change in the profiles along the horizontal direction and the tracking is not able to compensate for it.

Point‐to‐point dose difference plots were included to highlight the dose error (in cGy and %) as a function of distance across the profile. This allowed for a quantitative measure of dose errors and their locations, introduced by patient motion, and show the effectiveness of the tracking system in reducing areas of under and over dose. When motion was applied, there were under dose and over dose regions of up to 280 cGy (45% of max dose) and 200 cGy (35% of max dose) measured respectively, for a single fraction. The predictive tracking algorithm allowed the MLC to effectively compensate for the motion, the dose discrepancies were reduced to just 30 cGy which is 4.5% of the maximum dose. This represents a reduction in dose error of up to 90% when tracking is applied compared to the no‐motion case.

The effect of Patient 2 motion on the dose distribution is plotted in Figure 10. The motion profile is highly distorted with a triangular shape and large areas of under and over dose, up to ± 200 cGy. These dose errors are halved from 20% of maximum dose to around 10% when tracking is applied. There is not much effect from the motion along the horizontal component due to the small contribution of the motion in this direction.

Figure 11 shows the dose profile when Patient 3 motion trace was applied. Due to the predominantly left‐right component of this breathing pattern, the vertical profile is not largely affected; however, there is an overdose of 8% on the central axis in this direction when no tracking is applied which is reduced slightly to around 6% when tracking is activated.

The shift of the dose in the horizontal direction is evident in this case due to the large lateral component of the motion trace. Although the MP512 sensitive area is not large enough to reconstruct the full dose profile in this direction, the effect of motion causing distortion of the profile exemplifies the benefits of tracking.

## 5 CONCLUSIONS

CMRP has developed MP512, a 2D silicon diode array for use in small field dosimetry. It consists of 512 individual ion implanted diodes of size 0.5 × 0.5 mm^2^ with 2 mm center‐to‐center distance.

In this study, a two‐arc VMAT treatment was delivered to the phantom and MP512's angular dependence was corrected frame‐by‐frame. MP512 response was comparable to EBT3 film and the calculated TPS dose. These results provide confidence that detector‐based pretreatment dosimetry is clinically feasible despite fast real‐time MLC reconfigurations.

## CONFLICT OF INTEREST

The authors declare no conflict of interest.
